# Treatment of Long-Haul COVID Patients With Off-Label Acyclovir

**DOI:** 10.7759/cureus.37926

**Published:** 2023-04-21

**Authors:** Emily R German, Meera K Jairath, John Caston

**Affiliations:** 1 Psychiatry, Edward Via College of Osteopathic Medicine, Spartanburg, USA; 2 Psychiatry, Upstate Psychiatric Associates, Spartanburg, USA

**Keywords:** covid-19-related encephalopathy, covid-19 neurological outcomes, psychological issues due to covid-19, coronavirus disease (covid-19), covid long haul syndrome, long-haul covid, acyclovir therapy, covid 19

## Abstract

The SARS-CoV-2 virus (COVID-19) became a global pandemic in March 2020. This novel, highly infectious virus caused millions of infections and deaths around the world. Currently, there are few medications that are available for the treatment of COVID-19. Those affected are most commonly given supportive care, with some experiencing symptoms for months. We report a series of four cases depicting the successful use of acyclovir in the treatment of the virus SARS-CoV-2 in patients with long-haul symptoms, especially those in the realm of encephalopathy and neurological problems. Treatment with acyclovir in these patients resolved their symptoms and lowered their IgG and IgM titers, supporting the use of acyclovir as a safe and effective treatment for COVID-19 neurologic symptoms. We suggest the use of the antiviral medication, acyclovir, as a treatment for patients with long-term symptoms and unusual presentations of the virus, such as encephalopathy or coagulopathy.

## Introduction

The COVID-19 outbreak began in December 2019, and by March 2020, the World Health Organization announced that it had reached global pandemic status. This highly contagious and infectious disease created a public health crisis, infecting millions of people and causing a significant number of deaths [1].

COVID-19, also known as SARS-CoV-2, is a positive sense, single-stranded RNA virus leading to a range of illnesses in humans, from asymptomatic to life-threatening. Approximately 80% of infected people are asymptomatic or have mild symptoms [2]. The most common presenting symptoms of COVID-19 are fever (80-90%), cough (60-80%), and dyspnea (18-46%). Other symptoms include fatigue, myalgia, sore throat, nausea, vomiting, headache, diarrhea, anosmia, and impaired sense of taste [3,4].

COVID-19 primarily affects the respiratory system; however, its clinical presentation demonstrates that it can be a multi-system disease. It may affect the brain, causing delirium, seizures, or encephalopathy. In the heart, it may cause congestive heart failure, acute coronary syndrome, arrhythmias, or myocarditis. Kidney injury can result from systemic abnormalities. Skin abnormalities, including patchy erythematous rashes, have been reported as well [4].

Long COVID syndrome has been defined as signs or symptoms that continue to linger for over four weeks passed the initial infection. It is also described as being multi-systemic and may progress for months or years [5]. This is not on condition but a range with variable risks and outcomes. Some risk factors for developing long COVID syndrome include severe presentation of the disease or those were are not vaccinated against it [6]. Neurologic symptoms are one of the hallmark impairments in long COVID syndromes, including headache, vision changes, hearing loss, numbness in extremities, impaired mobility, tremors, memory loss, restless leg syndrome, sleep issues, impaired attention or difficulty concentrating (referred to as "brain fog"), and mood changes [7,8]. There have also been documented cases of patients developing COVID-induced encephalitis and/or encephalopathy [9]. The specific mechanisms of this pathology are poorly understood because of the novelty of the disease. Proposed mechanisms include a trans-nasal route from the olfactory nerve, external sources like intubation, or virus-specific factors [10].

Diagnosis of COVID-19 can be made qualitatively and quantitatively. The qualitative diagnostic test of choice is a reverse transcription polymerase chain reaction (RE-PCR) via nasal swab sample from the upper respiratory tract [3]. Alternatively, quantitative values of immunoglobulin antibodies can be used to monitor individuals’ immune response to the virus [11]. One useful way to measure COVID-19 immunity of individuals and communities is by quantifying the concentration of anti-receptor binding domain IgG antibodies, which is the specific antibody that targets the receptor binding domain of the S1 spike protein component of the SARS-CoV-2 virus [11,12].

Remdesivir is currently the only antiviral medication fully approved by the US FDA for the treatment of COVID-19 in both adults and pediatric patients (>28 days of age and >3 kg in weight) who test positive. This medication is approved for use in both hospital and non-hospital settings for those who are at increased risk for progressing to severe or life-threatening diseases [13]. Two other antiviral medications, Paxlovid (nirmatrelvir and ritonavir) and Lagevrio (molnupiravir), are approved for emergency use authorization due to the acute state of the pandemic [13]. Other drugs currently being used in treatment include hydroxychloroquine, lopinavir/ritonavir, oseltamivir, favipiravir, corticosteroids, and plasma from COVID-19 survivors [2]. Beyond these current medication therapies, there is no definitive treatment, and supportive care is the standard of care. Because remdesivir is an antiviral medication, it has been proposed that other antiviral medications, like acyclovir, may be able to target and treat COVID-19 infection.

Acyclovir acts by competitively inhibiting DNA polymerase through phosphorylation of the drug compound [14,15]. Acyclovir is now also being proposed to act by preventing the binding of IL-12 to the IL-12 receptor. This is promising for the treatment of COVID-19 by treating the high levels of serum cytokines, inhibiting the cytokine storm inflammatory response [16]. The use of acyclovir is FDA-indicated for the treatment of herpes zoster infections, genital herpes, varicella, and herpes simplex virus encephalitis [17,18]. Acyclovir has been used to treat COVID-19, with proven efficacy, safety, and low cost in small-scale studies [15].

The following case series supports the efficacy of acyclovir use in patients with unique long-haul COVID-19 symptoms. The purpose of this series is to demonstrate atypical presentations of COVID-19 and explore acyclovir as a treatment option in these patients.

## Case presentation

Patient A

In March 2021, Patient A, a 77-year-old male, presented in a psychiatric office with concerns about memory loss and agitation. The patient’s past medical history included anxiety, depression, and alcohol dependence disorder. He reported that he had tested positive for COVID-19 in December 2020. The patient’s wife described his hyperverbia, claiming “he has talked his head off to me.” IgG and IgM titers were obtained and were periodically drawn throughout the course of the disease progression. He returned to the same office in April 2021 with worsening complaints of memory loss, agitation, and hyperverbia. These symptoms and known COVID-19 infection led to the diagnosis of viral encephalopathy. This new diagnosis prompted treatment with acyclovir 400 mg three times daily (TID).

At the following appointments, the psychiatrist noted a flight of ideas and continued hyperverbia. The patient’s acyclovir dose was increased to 400 mg four times per day (QID) due to his persistent symptoms. In September 2021, the patient remarked that his agitation symptoms had resolved. The resolution of symptoms coincided with a trending decrease in IgG and IgM titers, with noted maintenance of spike antibodies >250 U/mL. Patient A presented with new complaints of generalized itchiness in January 2022 and was subsequently diagnosed with Rocky Mountain spotted fever (RMSF). This acute disease process was treated and resolved with doxycycline. During treatment for RMSF, acyclovir treatment was discontinued (February 2022). The psychiatrist continued to follow IgG and IgM titers for COVID-19 and noted in early June 2022 that the patient’s COVID-19 infection was likely resolved.

After receipt of the updated titers, it was found that the patient’s IgM had increased again, and the patient was restarted on acyclovir treatment with the goal to have IgM titers below 0.40 µg/ml. In July 2022, the patient had new complaints of nightmares and “doing crazy things,” symptoms indicative of encephalopathy. In August 2022, the patient was no longer hyperverbal but complained of an anger episode. The episode was later described as a fugue by a psychiatrist when the patient disclosed that he drove to a fire station in his boxer shorts. The patient also complained of a new infection with COVID-19, which was confirmed by IgG titer reaching >60 µg/ml at that time, as shown in Table 1.

**Table 1:** IgG and IgM quantitative values of Patient A The trends in the IgG and IgM titers (in μg/ml) for Patient A. IgG antibodies are used to show long-term immunity, while IgM is used to show acute response to the virus. The value of “60” in the table is any value >60, as 60 is the max value reported by the lab for IgG. The N/A values indicate that the Spike antibodies were not being tested at the time of these tests. Reference ranges for lab values obtained from the resulting agency Labtech Diagnostics.

	IgG (µg/ml)	IgM (µg/ml)	Spike (U/mL)
Normal	0.000-1.000 µg/ml	0.000-1.000 µg/ml	0.0-0.8 U/mL
Date			
3/19/21	0.717	0.8	N/A
5/14/21	60	5.072	>250
6/29/21	55.96	3.212	>250
7/21/21	33.79	2.038	>250
9/1/21	28.29	1.993	>250
10/6/21	21.41	1.617	>250
11/4/21	20.82	1.759	>250
12/14/21	13.47	0.963	>250
1/13/22	10.51	1.448	>250
2/15/22	8.402	0.923	>250
4/14/22	6.426	0.512	>250
5/16/22	6.351	0.517	>250
6/21/22	5.771	0.787	>250
7/6/22	5.826	0.739	>250
8/1/22	7.028	0.703	>250
8/22/22	60	0.962	>250
9/7/22	12.65	0.8	>250
9/28/22	14.93	0.25	>250

**Figure 1:**
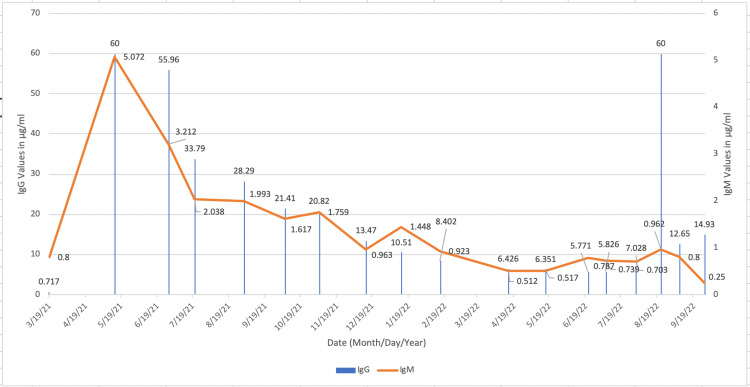
IgG and IgM trends in Patient A The trends in the titers for Patient A. A decreasing trend in both IgG and IgM indicates a decreasing immune response from the body as the patient recovers from the disease.

At the time of writing, acyclovir treatment is being continued at 400mg twice a day (BID) as symptoms resolve and the titers continue to trend downward (Figure 1).

Genetic testing on Patient A showed that he had no risk alleles for a weak IgG response. Because of this genetic composition, the patient had a very low chance of contracting COVID-19. For reference, the single nucleotide polymorphisms (SNPs) of this patient’s IgG response are FCGR2A His167Arg, GSTM3 V224I, and TNFRSF13B 16845167A>G. Due to the patient’s active and social lifestyle, he would have been hyper-exposed to the virus and susceptible to contracting it by proximity.

Patient B

Patient B is a 66-year-old male who presented to a psychiatry office in February 2021 for the main concern of memory loss. The patient had been experiencing memory loss for over a year, and it was affecting him at work. Patient B was married with two adult children, and both he and his spouse reported their first infection with COVID-19 in January 2021. The patient’s medical history included hyperthyroidism treated with irradiation and occlusion of the left internal carotid artery (ICA) with a filling defect in the anterior communicating artery, which was detected in computed tomography angiography (CTA) of the head and neck in February 2021. This CTA also showed evidence of old or current multifocal pneumonia, which was suggested to be embolic in origin. While initially complaining of memory loss, the patient’s chief complaints were found to be brain fog and confusion. At this initial psychiatric appointment, he was prescribed acyclovir 400 mg TID because of concern for viral encephalopathy (due to brain fog, confusion, and known COVID-19 infection) and increased coagulopathy.

The treatment was continued over the following months, with improvement seen in his IgG and IgM titers (Table 2).

**Table 2:** IgG and IgM quantitative values of Patient B The trends in the IgG and IgM titers (in μg/ml) for Patient B. IgG antibodies are used to show long-term immunity, while IgM is used to show acute response to the virus. The N/A values indicate that the spike antibodies were not being tested at the time of these tests. Reference ranges for lab values obtained from the resulting agency Labtech Diagnostics.

	IgG (µg/ml)	IgM (µg/ml)	Spike (U/mL)
Normal	0.000-1.000 µg/ml	0.000-1.000 µg/ml	0.0-0.8 U/mL
Date			
2/4/21	200	12.53	N/A
2/10/21	200	10.2	N/A
2/22/21	196.5	6.853	N/A
3/10/21	164.4	3.618	N/A
4/5/21	102.5	1.349	N/A
5/4/21	60	0.469	>250
8/4/21	20.16	0	>250
9/9/21	15.09	0	>250
11/3/21	9.882	0.076	>250

**Figure 2:**
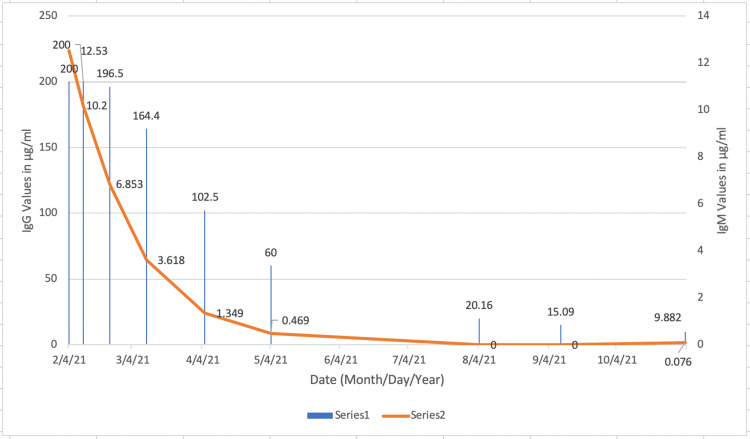
IgG and IgM trends in Patient B The trends in the titers for Patient B. A decreasing trend in both IgG and IgM shows a decreasing immune response from the body as the patient recovers from the disease.

There was a steady downward decreasing trend in his titers, as seen in Figure 2.

In March 2021, the patient was started on phosphatidylserine 100 mg BID as a supplement to further improve memory complaints [19]. In the following month, Patient B claimed resolution of his lack of focus. The psychiatrist then lowered his dose of acyclovir to 400 mg BID while continuing the phosphatidylserine supplements. In June, Patient B had further ICA ultrasound imaging which showed the same neurological findings as before. At this point, he asked whether to stop the acyclovir, but the psychiatrist recommended continuation until titers were within normal range.

Patient B and his spouse were both counseled to not receive vaccination for COVID-19 until their spike antibodies were below 75 (U/mL). As of September 2021, Patient B was on the continued treatment of acyclovir 400 mg BID due to the IgG titers but reported full resolution of symptoms and feeling better than he had in the past year. The patient will continue to follow up with his primary care physician.

Patient C

Patient C is a 66-year-old female who presented to a psychiatry office. This patient initially presented to the psychiatry office in 2016 with complaints of insomnia and short-term memory loss treated with phosphatidylserine. She lived with her husband and had one daughter.

In November 2020, the patient had headache symptoms and noticed her memory had gotten worse, which she believed to be due to the COVID-19 infection. At that time, she started 400 mg TID of acyclovir and recovered from her headache and memory symptoms. In December 2020, the patient started feeling ill again, with cough and viral respiratory symptoms lasting about a month, and then she took 15 more days of acyclovir 400 mg TID. She had a third resurgence of COVID-19 symptoms in April 2021 and restarted acyclovir 400 mg TID. From May to July 2021, the patient complained of increases in blood pressure and chest pain, which she had not previously experienced, and took acyclovir 400 mg TID continuously throughout this period. She presented with cough symptoms suggesting pneumonia; however, her CT scan of the chest results came back normal. In December 2021, the patient discontinued the use of acyclovir and reported the resolution of all previously experienced COVID-19 symptoms or sequelae. In 2022, the patient had no active COVID-19 infection and was no longer taking acyclovir. The patient had also received genetic testing for her COVID-19 genotypes, which showed that she has the IgG genotype consisting of an 83% propensity for decreased IgG synthesis. This could explain her abnormally low levels of IgG antibodies and spike levels, despite having been infected.

Patient C’s COVID-19 viral load was assessed by IgM and IgG titers (Table 3).

**Table 3:** IgG and IgM quantitative values of Patient C Trends in the IgG and IgM titers (in μg/ml) for Patient C. IgG antibodies are used to show long-term immunity, while IgM is used to show acute response to the virus. The N/A values indicate that the spike antibodies were not being tested at the time of these tests. Reference ranges for lab values obtained from the resulting agency Labtech Diagnostics.

	IgG (µg/ml)	IgM (µg/ml)	Spike (U/mL)
Normal	0.000-1.000 µg/ml	0.000-1.000 µg/ml	0.0-0.8 U/mL
Date			
11/24/20	0.018	0.2	N/A
12/10/20	0.255	0.905	N/A
2/3/21	1.687	2.079	N/A
2/11/21	1.281	2.112	N/A
2/26/21	0.905	2.194	N/A
3/17/21	0.644	0.988	N/A
4/9/21	0.353	2.289	57.1
5/4/21	2.107	1.947	61.2
6/4/21	1.768	1.418	74.7
7/21/21	0.64	0.959	60.7
9/1/21	0.82	0.961	68.6
10/15/21	0.673	0.688	70.1
11/16/21	0.577	0.808	64.5
12/17/21	0.547	0.766	63.5
1/20/22	0.567	0.723	58.8
3/11/22	0.513	0.677	62
5/16/22	0.377	0.402	59.5

**Figure 3:**
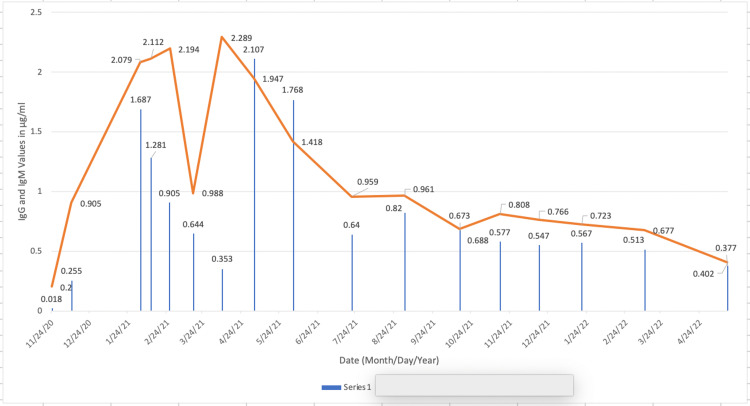
IgG and IgM trends in Patient C The trends in the titers for Patient C. A decreasing trend in both IgG and IgM shows a decreasing immune response from the body as the patient recovers from the disease.

IgM and IgG values titrated down (Figure 3), despite being low, while her spike antibody levels remained in the <80 (U/mL) range during treatment with acyclovir and once the patient stopped taking the medication.

A COVID-19 spike antibody less than 0.80 U/mL indicates a negative result for anti-SARS CoV-2S, which would suggest that this patient’s immune system had not generated a measurable response to the COVID-19 vaccination and that she has not had the COVID-19 infection [20]. This raised suspicion because the patient tested positive for COVID-19 despite her IgG and IgM antibody titers being only mildly elevated. When this information is considered alongside her genetic profile indicating she has a decreased propensity for making IgG despite infection, it may point to a weaker immune system.

Patient D

Patient D is a 38-year-old female who presented to a psychiatry office in 2021 and was diagnosed with anxiety, depression, dissociative disorder, functional neurological disorder, and unspecified abnormal involuntary movement disorder. The patient reported having sensory overstimulation episodes triggering a dissociative disorder with trance-like episodes. The patient had an older sister and mother who lived in the area, and she was married and had two daughters. She worked at a wholesale store and had a good support system in the area.

In late February 2021, the patient acquired a COVID-19 infection and complained of experiencing intense body heat, without sweating, needing four fans in the bedroom to help alleviate symptoms. She was started on 400 mg of acyclovir BID and continued taking it until December 2021. After 10 months of this treatment regimen, she was in remission. The patient did not experience any other multi-system effects from the virus.

This patient’s COVID-19 viral load was assessed by IgM and IgG titers (Table 4).

**Table 4:** IgG and IgM quantitative values of Patient D The trends in the IgG and IgM (in μg/ml) titers for Patient D. IgG antibodies are used to show long-term immunity, while IgM is used to show acute response to the virus. The value of “60” in the table is any value >60, as 60 is the max value reported by the lab for IgG. The N/A values indicate that the spike antibodies were not being tested at the time of these tests. Reference ranges for lab values obtained from the resulting agency Labtech Diagnostics.

	IgG (µg/ml)	IgM (µg/ml)	Spike (U/mL)
Normal	0.000-1.000 µg/ml	0.000-1.000 µg/ml	0.0-0.8 U/mL
Date			
2/17/21	0.022	0.562	N/A
2/25/21	7.673	0.963	N/A
5/3/21	23.72	0.963	>250
8/10/21	9.893	0.496	>250
9/7/21	9.477	0.204	>250
11/1/21	7.882	0.514	>250
12/30/21	7.49	0.716	>250
3/17/22	60	0.159	>250

**Figure 4:**
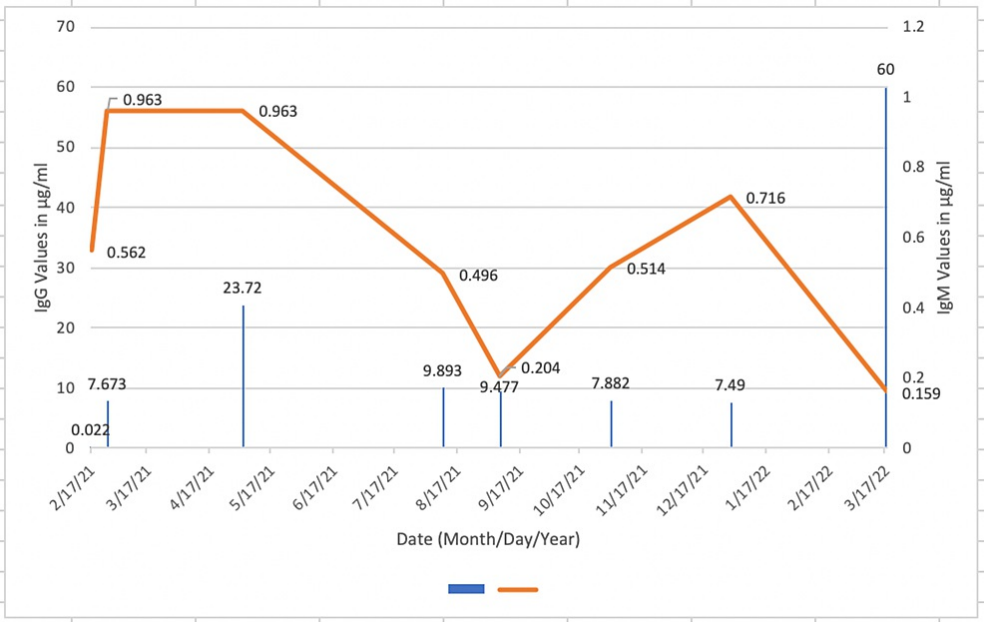
IgG and IgM trends in Patient D The trends in the titers for Patient D. A decreasing trend in both IgG and IgM shows a decreasing immune response from the body as the patient recovers from the disease.

Her IgM and IgG antibody values titrated down, while her spike antibody levels remained >250 (U/mL) (Figure 4).

An IgG level of >60 (µg/ml) measured on March 17, 2022, indicated re-infection with COVID-19, suggestive of a new variant. Of note, Patient C in our case series is the mother of this patient (Patient D). Patient D’s IgG levels were much higher than her mother’s, indicating genetic variation between the two patients’ IgG profiles.

Patient D’s allelic profile showed no risk alleles for a weak IgG response, in contrast with her mother’s (Patient C). For reference, the SNPs of this patient's IgG response were FCGR2A His167Arg, GSTM3 V224I, and TNFRSF13B 16845167A>G. On the other hand, her mother, Patient C’s SNPs were FCGR2A and GSTM3 V224I, with the A risk allele on the FCGR2A SNP, indicating an association with a lower IgG response and with COVID-19 infection.

## Discussion

The multi-system effects of COVID-19, specifically encephalopathy, coagulopathy, and lasting lung damage, have been identified in patients with long COVID syndrome. There are multiple drugs in clinical trials for the treatment of this condition. However, there is no current FDA medication approved to treat COVID-19 encephalopathy specifically.

In this case series, the physician decided the use of acyclovir off-label to treat Patient A-D because of the limited options available for treating long COVID syndrome. The dosing for these patients was acyclovir tablets taken by mouth BID, TID, and QID. The physician closely monitored the patients’ progress by quantitatively assessing the immunoglobulin titer values and symptoms as reported by the patients. Treatment was deemed successful based on continuously decreasing immunoglobulin values IgG and IgM, as well as patients’ reported improvement of symptoms.

Acyclovir was first discovered in 1974 and became available for physician use in 1982 [21]. It is generally considered to be a safe and efficacious drug for viruses such as herpes viruses, varicella zoster, and cytomegalovirus. The drug has little to no effect on healthy cells, targeting only those affected [21]. It is predominantly excreted via the kidneys. Thus, caution must be used when prescribing to those with kidney disease [18]. The only absolute contraindication with the use of acyclovir is hypersensitivity to the drug [18]. The most common adverse effect of the drug is a general malaise, while less commonly there can be abdominal pain, agitation, dizziness, anemia, or fatigue [18]. Overall, acyclovir is well-tolerated in most patients, with few side effects if any. None of the patients in this case series reported side effects from the medication.

Each of the patients in this case series presented with unique symptoms. Patient A had the peculiar symptom of hyperverbia. This patient had not previously been noted to be particularly talkative, and after infection with COVID-19, he was not able to slow down his talking speed. This symptom is not commonly reported and shows the range of effects the COVID-19 virus can have on the brain. Patient B had embolic changes suspected and evidence of pneumonia on imaging. He had many symptoms consistent with viral encephalopathy, a diagnosis supported by image findings. Patients C and D were intriguing due to their genetic differences and relationship as a mother/daughter pair. Patient C had a weakened immune system, and her titer values were novel to follow. Genetically, Patient D did not have this weaker immune response, and her lab values corroborated. Of note, Patient D also had the uncommon symptom of intense body heat without sweating.

Limitations

While this case series presents positive outcomes for COVID-19 encephalopathy symptoms, there are several limitations. The series presents four presentations of atypical, unique symptoms of COVID-19. This sample size is important to note in that the generalizability to larger populations is unclear. A second notable limitation is that while symptoms reportedly improved and IgG/IgM titers decreased, it is not known whether this is a direct response from acyclovir treatment or from natural disease resolution. Another limitation would be that some of the patients discussed had concerns about memory prior to known COVID-19 infection. Further studies, including randomized control studies with larger populations, are needed in the future to confirm the therapeutic benefits of acyclovir for COVID-19 encephalopathy. It would also be beneficial for future studies to complete imaging including CT or MRI of the brain to evaluate structural differences with treatment, as well as consult with other physicians including neurologists and pulmonologists.

## Conclusions

While the COVID-19 virus is known to predominantly affect the respiratory system, the neurological effects of viral encephalopathy for this virus are less known. Treatment for COVID-19 encephalopathy is limited, and no drug has been FDA-approved for this use specifically. The patients described in this case series depict unique and uncommon symptoms of encephalopathy after the contraction of the COVID-19 virus. Treatment with acyclovir in these patients may have resolved their symptoms and lowered their IgG and IgM titers. Future randomized control studies will be needed to show acyclovir's effects on COVID-19, as well as rule out symptom resolution due to the natural progression of the disease. The patients in this series did not complain of medication side effects and have not reported a return of their symptoms since completing their treatments. This case series suggests that the use of acyclovir may be a safe and effective treatment for COVID-19 neurologic symptoms in the future.
